# Molecular basis of resistance to macrolides, lincosamides and streptogramins in *Staphylococcus hominis* strains isolated from clinical specimens

**DOI:** 10.1007/s12223-015-0419-6

**Published:** 2015-08-09

**Authors:** Ewa Szczuka, Nicoletta Makowska, Karolina Bosacka, Anna Słotwińska, Adam Kaznowski

**Affiliations:** Department of Microbiology, Institute of Experimental Biology, Faculty of Biology, Adam Mickiewicz University, ul. Umultowska 89, 61-614 Poznań, Poland; Department of Microbiological and Laboratory Diagnostics, Bacteriological Laboratory, Regional Hospital in Poznań, Juraszów 7/19, 60-479 Poznań, Poland; Bacteriological Laboratory, Regional Hospital in Kołobrzeg, E. Łopuskiego 33, 78-100 Kołobrzeg, Poland

## Abstract

Coagulase-negative staphylococci (CoNS) are the most frequently isolated bacteria from the blood and the predominant cause of nosocomial infections. Macrolides, lincosamides and streptogramin B (MLS_B_) antibiotics, especially erythromycin and clindamycin, are important therapeutic agents in the treatment of methicillin-resistant staphylococci infections. Among CoNS, *Staphylococcus hominis* represents the third most common organism. In spite of its clinical significance, very little is known about its mechanisms of resistance to antibiotics, especially MLS_B_. Fifty-five *S. hominis* isolates from the blood and the surgical wounds of hospitalized patients were studied. The *erm*(C) gene was predominant in erythromycin-resistant *S. hominis* isolates. The methylase genes, *erm*(A) and *erm*(B), were present in 15 and 25 % of clinical isolates, respectively. A combination of various erythromycin resistance methylase (*erm*) genes was detected in 15 % *S. hominis* isolates. The efflux gene *msr*(A) was detected in 18 % of isolates, alone in four isolates, and in different combinations in a further six. The *lnu*(A) gene, responsible for enzymatic inactivation of lincosamides was carried by 31 % of the isolates. No erythromycin resistance that could not be attributed to the genes *erm*(A), *erm*(B), *erm*(C) and *msr*(A) was detected. In *S. hominis*, 75 and 84 %, respectively, were erythromycin resistant and clindamycin susceptible. Among erythromycin-resistant *S. hominis* isolates, 68 % of these strains showed the inducible MLS_B_ phenotype. Four isolates harbouring the *msr*(A) genes alone displayed the MS_B_ phenotype. These studies indicated that resistance to MLS_B_ in *S. hominis* is mostly based on the ribosomal target modification mechanism mediated by *erm* genes, mainly the *erm*(C), and enzymatic drug inactivation mediated by *lnu*(A).

## Introduction

Coagulase-negative staphylococci (CoNS) are part of the normal bacterial flora of human skin, but they have been increasingly recognized as opportunistic pathogens capable of causing various types of infections (Piette and Verschraegen 2009). Among clinically significant strains of CoNS, *Staphylococcus hominis* is ranked the third in importance only after *S. epidermidis* and *S. haemolyticus.* The *S. hominis* is a genetically diverse species, and it is believed that recombination plays a significant role in generating this diversity (Mendoza-Olazarán et al. 2013; Zhang et al. 2013; Szczuka et al. 2014). These bacteria can be responsible for blood stream infections, endocarditis, peritonitis, bone and joint infections (Kloos and Bannerman 1999; Kaufman and Fairchild 2004; Chaves et al. 2005; Sorlozano et al. 2010; Bouchami et al. 2011). Similar to other staphylococci, the formation of biofilm on medical devices, or on host tissues, is thought to be the one of the major pathogenic factors of *S. hominis* (Kaufman and Fairchild 2004; Götz et al. 2006; Chokr et al. 2006; Rodhe et al. 2006; Fredheim et al. 2009; Szczuka et al. 2015). Relatively high prevalence of methicillin resistance complicated the treatment of staphylococcal infections (Casey et al. 2007). Macrolides, lincosamides and streptogramin B antibiotics are the preferred alternative to penicillins and cefalosporins in the treatment of staphylococci infection. Moreover, erythromycin and clindamycin are recommended as second-line drugs for patients with a β-lactam allergy (Leclercq 2002; Gherardi et al. 2009). MLS_B_ antibiotics are structurally distinct but functionally similar because they inhibit protein synthesis by binding to the 50S subunit (23S rRNA) of the bacterial ribosome. In staphylococci, resistance to MLS_B_ is generally based on three mechanisms: the ribosomal target modification mediated by *erm* genes, the active efflux of antibiotics mediated by *msr*(A) and enzymatic drug inactivation mediated by *lnu*(A) (Leclercq 2002). The *lnu*(A) gene encodes lincosamide O-nucleotidyltransferase, which only inactivates lincosamides. Erythromycin-resistance methylase (*erm*) genes encode proteins which methylate adenine residue A2058 in the peptidyltransferase region of 23S rRNA domain V, which is part of the large (50S) ribosomal subunit and prevents the binding of the antibiotic to the target site (Leclercq 2002; Novotna et al. 2005). This methylation results in cross-resistance to macrolide, lincosamide and streptogramin B antibiotics (MLS_B_ phenotype), which can be expressed either constitutively (cMLS_B_) or inducibly (iMLS_B_). Coagulase-negative staphylococci, with an iMLS_B_ resistance phenotype are resistant to 14-membered and 15-membered macrolides, whereas CoNS with a cMLS_B_ resistance phenotype are resistant to all MLS_B_ antimicrobials. The *msr*(A) gene is involved in the active efflux of antibiotics, causing resistance to 14- and 15-membered macrolides as well as to streptogramin, but not to lincosamides (MS_B_ phenotype). This makes clindamycin, as a treatment choice, effective (Lina et al. 1999; Leclercq 2002; Vimberg et al. 2015).

The main purpose of this study was to assess the molecular basis of resistance to MLS_B_ antibiotics in clinical isolates of *S. hominis*.

## Material and methods

### Bacterial strains

Fifty-five isolates of *S. hominis* were collected from the blood and surgical wound swabs of hospitalized patients. True bacteremia was diagnosed in 36 the of patients. The isolates were identified by using the VITEK 2 system (bioMérieux, France). Although the *tuf* sequencing gives perfect results in the identification of this species, the VITEK 2 offers very good results as well. Because the *S. hominis* is a genetically diverse species, we confirmed the identification of all tested *S. hominis* isolates by using the API STAPH. In this study, we included only those isolates whose identification was beyond any doubt. The isolates were stored at −70 °C, in 50 % glycerol broth (BHI), until commencement of the study.

### Characterization of resistance mechanisms

Phenotypic characterization of macrolides and lincosamides resistance was determined by the double-disc test, with erythromycin (15 μg) and clindamycin (2 μg) discs applied 20 mm apart. A 10-μl inoculum of a 0.5 McFarland suspension was spotted on Mueller-Hinton agar with antibiotic disc. After 18 h incubation at 35 °C, blunting of the clindamycin zone of inhibition proximal to the erythromycin disc indicated the inducible type (D-shaped zone) of MLS_B_ resistance, whereas resistance to both erythromycin and clindamycin indicated the constitutive type. Lack of a D-shaped zone in erythromycin-resistant and clindamycin-susceptible isolates was interpreted as the MS_B_ efflux phenotype (Leclercq 2002; Aktas et al. 2007). The results were interpreted according to EUCAST recommendations. Isolates were also screened with a 30-μg cefoxitin disc and studied for the presence of *mecA* genes to test methicillin resistance (Geha et al. 1994). The bacterial genomic DNA was isolated from clinical isolates using the Genomic DNA Plus kit (A&A Biotechnology, Poland). For the detection of macrolide resistance genes (*erm*(A)*, erm*(B), *erm*(C), *msr*(A), *lun*(A)) and *mecA* genes, PCR assays were performed as described by Lina et al. (1999), Le Bouter et al. (2011) and Geha et al. (1994). The STATISTICA software (10.00 StatSoft, Tulsa, OK, USA) was used for statistic analysis. Association between methicillin resistance and resistance to MLS_B_ antibiotics was evaluated by using chi-square (*χ*^2^) test. A *P* value of <0.05 was considered significant.

## Results

The most prevalent resistance determinant was *erm*(C) which was detected in 25 of the isolates (45 %), followed by *lnu*(A), *erm*(B) and *erm*(A) detected in 17 (31 %), 14 (25 %) and 8 (15 %) isolates, respectively. The *msr*(A) gene was detected alone, in 4 isolates and in 6 isolates, in combination with other genes. As Table 1 shows, 14 distinct resistance genotypes could be observed in the *S. hominis* strains. Fourteen isolates were negative for all screened genes.

**Table 1:** Distribution of resistance genes *erm*A, *erm*B, *erm*C, *msr*A and *lin*A among *S. hominis* clinical strains

Resistance genotype	No. of isolates	No. of isolates with phenotype		
		MLS_B_—inducible	MLS_B_—constitutive	MS_B_
*erm*B	1	1	0	0
*erm*C	9	9	0	0
*msr*A	4	0	0	4
*erm*A+ *erm*B	3	3	0	0
*erm*A+ *erm*B+ *erm*C	2	2	0	0
*erm*B+ *msr*A	2	2	0	0
*erm*C+ *msr*A	2	2	0	0
*erm*A+ *lnu*(A)	1	0	1	0
*erm*B+ *lnu*(A)	2	0	2	0
*erm*C+ *lnu*(A)	10	5	5	0
*erm*A+ *erm*B+ *lnu*(A)	2	1	1	0
*erm*B+ *erm*C+ *lnu*(A)	1	1	0	0
*erm*B+ *msr*A+ *lnu*(A)	1	1	0	0
*erm*C+ *msr*A+ *lnu*(A)	1	1	0	0
No gene	14	0	0	0
Total	55	28	9	4

All isolates harbouring the *erm*(B) or *erm*(C) genes alone or in combination with other genes exhibited resistance to erythromycin. The *erm*(A) was never found alone and all *erm*(A)-positive isolates were resistant to erythromycin. Fourteen isolates, which were negative for all five resistance genes, displayed susceptibility to erythromycin and clindamycin. No isolates, resistant to clindamycin only, were found. Twenty eight *erm*-positive isolates were resistant to erythromycin but remained susceptible to clindamycin and exhibited the inducible MLS_B_ phenotype. The remaining nine *erm-*positive isolates showed resistance to erythromycin and clindamycin, displaying the constitutive MLS_B_ phenotype. It should be emphasized that the cMLSB phenotype was detected only in strains harbouring simultaneously *erm* and *lnu*(A). The four isolates, harbouring the *mrsA* gene alone, represented the MS_B_ phenotype. Methicillin-resistant *S. hominis* isolates were significantly more often resistant to macrolides and lincosamides (93 % to erythromycin, 77 % to clindamycin) than methicillin-susceptible isolates (50 and 22 %, respectively; *p* < 0.001).

## Discussion

Coagulase-negative staphylococci have been recognized as an important cause of nosocomial infections and are the most frequently isolated bacteria from blood (Krediet et al. 2004; Hira et al. 2007; Piette and Verschraegen 2009). These pathogens have developed an increased resistance to antimicrobial agents, especially to methicillin and other semisynthetic penicillins. Among CoNS, *S. haemolyticus* has the highest tendency to develop resistance to multiple antibiotics (Rodríguez-Aranda et al. 2009). *S. hominis* isolates display a lower virulence than *S. haemolyticus* and have been recognized, less frequently, as significant human pathogens. However, there are reports indicating that *S. hominis* can be responsible for nosocomial outbreaks (Chaves et al. 2005; d’Azevedo et al. 2008; Palazzo et al. 2008; Sorlozano et al. 2010; Ruiz de Gopegui et al. 2011; Roy et al. 2014). Nevertheless, there is limited information on their resistance to antibiotics, especially to macrolides, lincosamides and streptogramin B. As mentioned above, MLS_B_ are used against staphylococcal infection in penicillin-allergic patients and in methicillin-resistant staphylococci (MRS)-infected patients. In particular, the use of clindamycin is regarded as a valid choice in the treatment of soft-tissue and bone infections (Lina et al. 1999; Leclercq 2002; Gherardi et al. 2009). The present data indicates that 16 % of *S. hominis* strains were resistant to clindamycin, whereas 75 % displayed resistance to erythromycin. In German studies, only 19 % of *S. hominis* strains were erythromycin resistant (Gatermann et al. 2007). Most of these strains displayed the constitutive MLS_B_ phenotype, as opposed to our study, which demonstrated that the majority of *S. hominis* expressed the inducible MLS_B_ phenotype. It should be emphasized, that coagulase-negative staphylococci, with an iMLS_B_ resistance phenotype are resistant to 14-membered and 15-membered macrolides, but susceptible to lincosamides, streptogramin B and 16-membered macrolides. Although, iMLS_B_ CoNS are in vitro resistant to erythromycin and in vitro sensitive to clindamycin, prescribing clindamycin may lead to treatment failure. In our studies, more than half of the *S. hominis* isolates were resistant to methicillin. Additionally, methicillin resistance was closely associated with resistance to erythromycin, which narrows the therapeutic options. It is well known that glycopeptides are the treatment of choice for infections caused by the multi-resistant staphylococci. However, due to the emergence of vancomycin-resistant staphylococci, a reduction in the use of this antibiotic has been recommended. Recently, Won and Kim (2013) has reported the emergence of vancomycin-resistant *S. hominis.* Also, the emergence of resistance to relatively new antibiotics, such as linezolid and quinupristin/dalfopristin, has also been noted, in clinical *S. hominis* strains (Petinaki et al. 2005; Ruiz de Gopegui et al. 2011).

This study indicated that the resistance to macrolides and lincosamides in *S. hominis* is mostly based on the ribosomal target modification mechanism mediated by *erm* genes; mainly the *erm*(C) and enzymatic drug inactivation, mediated by *lnu*(A). The *erm*(C) genes are predominant among coagulase-negative staphylococci from European countries, Canada and Korea (Martineau et al. 2000; Lim et al. 2002; Novotna et al. 2005; Gatermann et al*.*^2007^; Gherardi et al. 2009). However, these data largely concerns the most frequently isolated coagulase-negative strains i.e. *S. epidermidis* and *S. haemolyticus*, whereas little is known about the distribution of MLS_B_ resistance genes in other staphylococci species, including *S. hominis.* Recently, Le Bouter et al*.* (2011) characterized resistance to macrolides, lincosamides and streptogramin B in 72 *S. saprophyticus* strains isolated from urine specimens. They found that the distribution of MLS_B_ resistance genes in *S. saprophyticus* is different from that generally reported for *S. epidermidis* and *S. haemolyticus.* The results of this study show that *erm*(A) and *erm*(B) genes were present more frequently in *S. hominis* than in other staphylococcal species as previously described (Martineau et al. 2000; Gatermann et al. 2007; Gherardi et al. 2009). For example, in a study conducted in Korea, e*rm*(B) genes were present only in 3.3 % of isolates (Lim et al. 2002). The efflux of macrolides due to *msr*(A) is a mechanism found only in a minority of *S. hominis*. Previously obtained data indicates that *msr*(A) genes were present in 11–24 % of coagulase-negative staphylococci (Aktas et al. 2007; Bouchami et al. 2007; Gatermann et al. 2007). In contrast, in *S. saprophyticus*, the efflux mechanisms were the most common mechanisms of resistance to MLS_B_ antibiotics (Le Bouter et al. 2011). We observed a high occurrence of the *lnu*(A) gene, which confer resistance to lincomycin, but clindamycin remains active (Leclercq 2002). Overall, this study suggested that *S. hominis* may constitute a reservoir for MLS_B_ genes, in particular *erm*(C) and *lnu*(A), among coagulase-negative staphylococci. These resistance genes are often located on plasmids or transposons and may be transferable to more pathogenic staphylococcal species (Leclercq 2002).

Our results indicated that the uncommon pathogen, *S. hominis* had a high prevalence of erythromycin resistance and most of these strains display the inducible MLS_B_ phenotype. Ribosomal modification and drug inactivation are the main mechanisms of MLS_B_ resistance, in *S. hominis* strains*.*
